# Delayed proximal hamstring tendon repair after ischial tuberosity apophyseal fracture in a professional volleyball athlete: a case report

**DOI:** 10.1186/s12891-021-04468-2

**Published:** 2021-06-24

**Authors:** Patricia M. Lutz, Michel Knörr, Stephanie Geyer, Andreas B. Imhoff, Matthias J. Feucht

**Affiliations:** 1 Department for Orthopedic Sports Medicine, Technical University Munich, Ismaninger Str. 22, 81675 Munich, Germany; 2 Department of Anaesthesiology, University Hospital, LMU Munich, Munich, Germany; 3 Department of Orthopedics and Trauma Surgery, Medical Center, Faculty of Medicine, Albert-Ludwigs-University of Freiburg, Freiburg, Germany

**Keywords:** Ischial tuberosity apophyseal fracture, Hamstring tendon repair, Avulsion fracture, Hamstring injury, Case report

## Abstract

**Background:**

Ischial tuberosity apophyseal fractures are avulsion fractures of the anatomic footprint of the proximal hamstring tendons. Generally, these injuries are rare and frequently occur in skeletally immature, active patients due to incomplete ossification. Depending on the fragment displacement, non-operative or operative treatment approaches are used.

**Case presentation:**

We report a case of a 29-year-old professional volleyball athlete who has suffered from a nonunion avulsion fracture for 14 years. Isolated suture anchor fixation was performed after open excision of a large bony fragment followed by excellent clinical and functional outcome at 1 year postoperatively.

**Conclusion:**

In conclusion, avulsion fractures of the ischial tuberosity with large fragments and restrictions to activities of daily living due to pain can, in individualized cases, be treated with an open excision of the fragment followed by repair of the proximal hamstring tendons using suture anchors.

## Background

Ischial tuberosity apophyseal fractures are avulsion fractures of the anatomic footprint of the proximal hamstring tendons [1]. Generally, these injuries are rare and frequently occur in skeletally immature, active patients due to incomplete ossification [1–3]. Depending on the displacement of the fragment, nonunion after conservative treatment, or sciatic nerve complications, an operative excision or refixation of the fragment with combined repair of the hamstring tendons should be considered [2, 4–6]. Multiple surgical approaches have been published to surgically treat avulsion fractures with proximal hamstring injuries including reconstruction plates, lag screws, cancellous screws, and suture anchors [5–10].

We report a case of a 29-year-old professional volleyball athlete who has suffered from a nonunion avulsion fracture for 14 years due to delayed diagnosis. We performed an open excision of a large fragment followed by isolated suture anchor fixation of the proximal hamstring tendons.

## Case presentation

A 29-year-old healthy male, who was a former professional volleyball player, presented himself in our outpatient clinic. The patient reported of a delayed diagnosis (3 years after initial trauma) of a proximal hamstring injury due to a soccer injury as a 15-year-old adolescent. Similar to previous case reports [11], the patient reported of numerous previous consultations with physiotherapists, general practitioners, and sports physicians. A gradual increase in daily pain underneath the buttock with any kind of sports, longer walking, and while sitting led to a pronounced desire for surgical therapy. Additionally, increased impairment in the athlete’s performance with muscle weakness of the hamstrings accentuated by hamstring stretching led to severe restrictions in the patient’s daily life. The patient’s family history and previous medical history were unremarkable. The initial physical examination revealed a significant tenderness along the proximal hamstring tendons and at the ischial tuberosity with no clear palpable defect at the hamstring insertion at the ischial tuberosity. Active and passive range of motion (ROM) of hip and knee were unremarkable. No neurological deficit was present, but a slight hamstring strength deficit (4/5) was evident. Evaluation of the contralateral limb was normal.

**Fig. 1:**
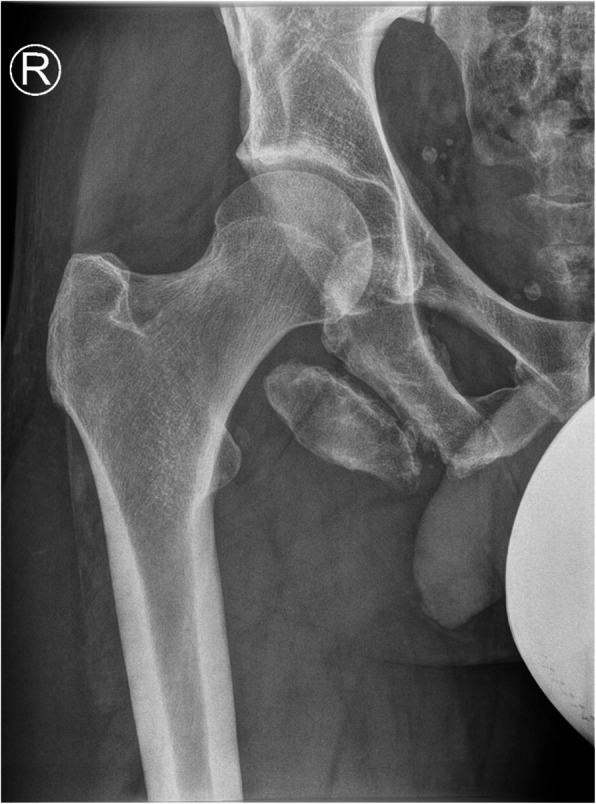
Preoperative radiograph showed right ischial tuberosity apophyseal fracture with a large displaced fragment

**Fig. 2:**
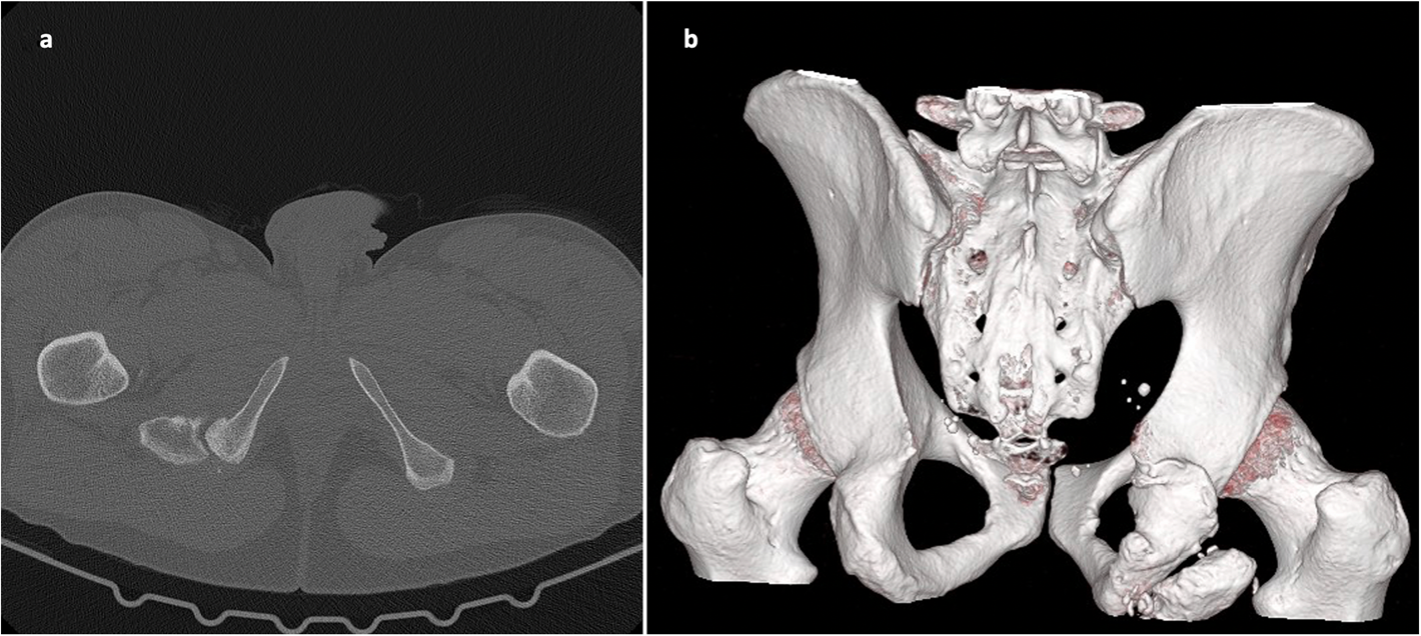
Preoperative CT showed nonunion of the displaced fragment (**a**) and location of the fragment in preoperative three-dimensional CT (**b**)

**Fig. 3:**
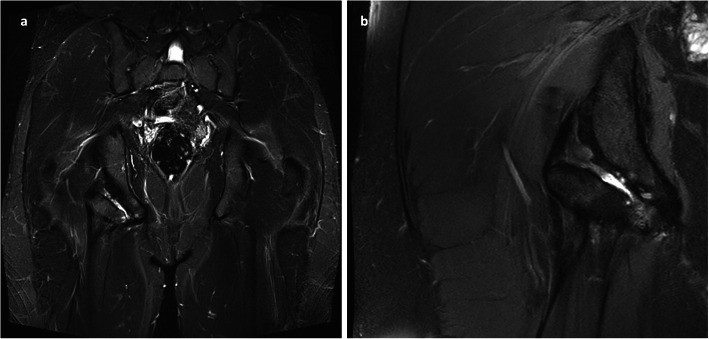
Preoperative MRI showed right anatomic footprint of hamstring tendons displaced from the ischial tuberosity (**a**, **b**)

Plain radiographs (Fig. 1) and computed tomography (CT) of the pelvis showed an ischial tuberosity apophyseal fracture with a 7 × 3 centimeter (cm) large, displaced fragment (< 1 cm) (Fig. 2). Magnet resonance imaging (MRI) of the pelvis showed nonunion and displacement of approximately 1 cm, with fluid accumulation between the avulsed fragment (Fig. 3). All proximal hamstring tendons were attached to the avulsed fragment.

Due to daily complaints we decided to try to combine the excision of the large bony fragment with repair of the proximal hamstring tendons with the risk of not being able to re-attach the tendons at the ischial footprint because of the delayed diagnosis.

### Surgical intervention

**Fig. 4:**
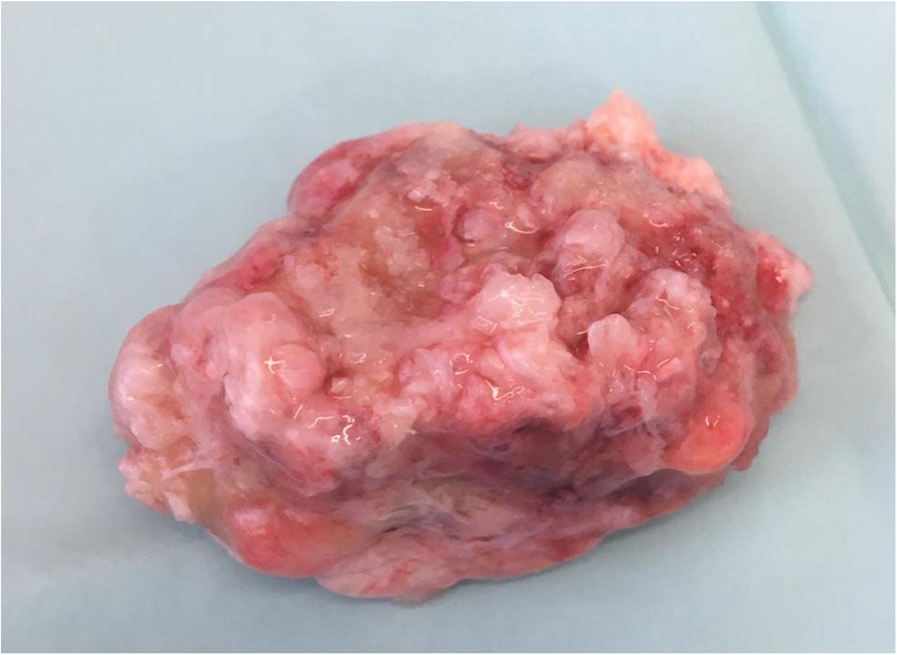
Large bony fragment after open excision and debridement

**Fig. 5:**
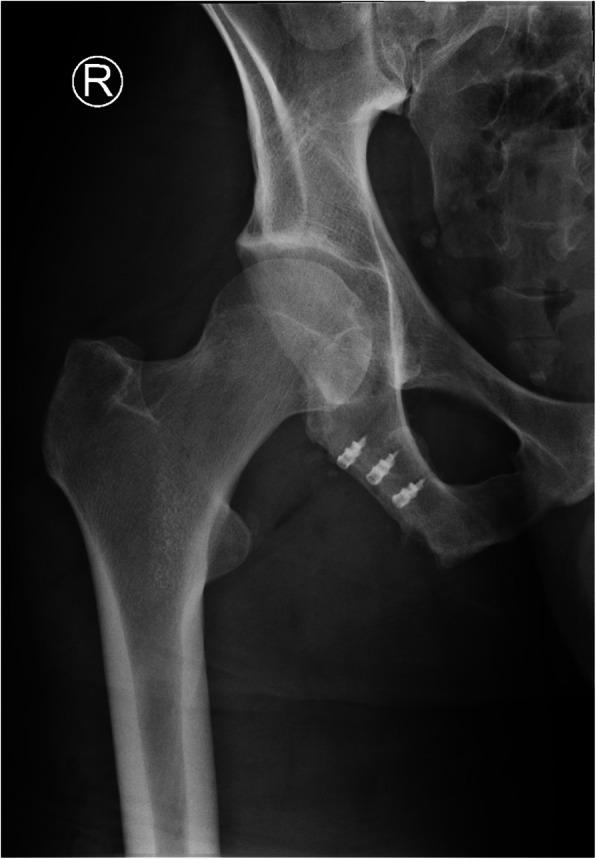
Postoperative radiograph showed correct position of three suture anchors and total excision of the nonunion fragment

Fourteen years after the initial trauma, an open excision of the large bony fragment (Fig. 4) and an open repair of the proximal hamstring tendons was performed. The patient was placed in a prone position. A 10 cm incision was made in the gluteal fold, and a subgluteal approach was performed. With special attention to the sciatic nerve, the large bony fragment was identified and an excision, followed by debridement of local fibrous tissue and mobilization of the hamstring tendons, was performed. To reduce excessive strain on the tendons, the mobilization was extended distally. Then refixation of the proximal hamstring tendons was performed with 3 suture anchors (Titan-Corkscrew 5.5 mm (mm), FiberWire, Arthrex, Naples, USA) at the ischial tuberosity. Radiographs confirmed the correct position of the anchors (Fig. 5).

### Postoperative course

Postoperatively, the patient was mobilized on crutches without load for 6 weeks. A hard frame hip and knee brace (Newport, DJO, Lewisville, USA) fixed in 30° of hip flexion was used for 6 weeks (24 h per day). After six postoperative weeks, full weight-bearing was allowed and a supervised physical therapy program was started for 12 weeks. Nine months postoperatively, the patient had returned to playing volleyball on a semi-professional level. Twelve months postoperatively, the patient was extremely satisfied with the surgical outcome and was able to perform all kind of desired sport activities (volleyball, beachvolleyball, hiking, kite-surfing, climbing, and skiing) and activities of daily living without difficulty or pain. At final follow-up, Perth Hamstring Assessment Tool [12] was 94/100 points.

## Discussion

This case report describes an athlete suffering from an ischial avulsion fracture for many years. An open excision of a large fragment was combined with isolated suture anchor repair of proximal hamstring tendons.

Apophyseal fractures of the ischial tuberosity generally occur in young athletes during activities, where hamstring muscles are contracted and the leg is forced into hyperflexion of the hip with full extension of the knee [2, 7, 13]. Conservative treatment is widely accepted if the fragment displacement is less than 1.5 cm [1, 4]. Surgical treatment is only recommended in the case of nonunion, fibrosis, muscle weakness, nerve complications, if pain occurs, or if the displacement is greater than 1.5 cm [2, 4]. Lempainen et al. [14] investigated an individualized muscle-tendon concept in athletes and concluded that complete proximal hamstring avulsions have a poor prognosis to heal without operative treatment and should therefore be managed surgically in top-level athletes. Multiple surgical approaches have been published to surgically treat avulsion fractures with proximal hamstring injuries including reconstruction plates, lag screws, cancellous screws, and suture anchors [5–10]. A delay in surgical repair of hamstring tendons may increase the likelihood of sciatic nerve involvement, reduces postoperative outcome concerning strength and endurance, and increases the risk of postoperative complications [13, 15]. Skaara et al. [16] stated that nearly 60% of patients returned to preinjury activity levels and Willinger et al. [15] reported a high return to sport rate (86%) after repair of proximal hamstring avulsions. In previous research by McGregor et al. [11], only 35% of patients had returned to sport activities at their pre-injury level after explorations with limited debridement and suture of the tendon.

Independent of the extent of dislocation or the delay of diagnosis, however, in the present case, an open excision of the fragment followed by isolated suture anchor repair of the hamstrings was performed. Although the delayed diagnosis made it technically more challenging, excellent clinical, functional, and subjective results were achieved 1 year postoperatively.

In conclusion, avulsion fractures of the ischial tuberosity with large fragments and restrictions to activities of daily living due to pain can, in individualized cases, be treated with an open excision of the fragment followed by repair of the proximal hamstring tendons using suture anchors.

## Data Availability

The datasets used and analyzed during the current case report are available from the corresponding author on reasonable request.

## Abbreviations

cm: Centimeter
CT: Computed tomography
mm: Millimeter
MRI: Magnet resonance imaging
ROM: Range of motion
